# Towards understanding governance issues in integration of mental health into primary health care in Uganda

**DOI:** 10.1186/s13033-016-0057-7

**Published:** 2016-03-24

**Authors:** James Mugisha, Joshua Ssebunnya, Fred N. Kigozi

**Affiliations:** EMERALD Project, Butabika National Referral and Teaching Hospital, P.O. Box 7017, Kampala, Uganda; Kyambogo University, Kampala, P.O. Box 1 Kyambogo, Kampala, Uganda

**Keywords:** Governance, Integration, Mental health, PHC, Uganda

## Abstract

**Background:**

There is a growing burden of mental illness in low income countries. The situation is further worsened by the high poverty levels in these countries, resulting in difficult choices for their health sectors as regards to responding to the burden of mental health problems. In Uganda, integration of mental health into primary health care (PHC) has been adopted as the most vital strategy for ensuring mental health service delivery to the general population.

**Objectives:**

To identify governance related factors that promote/or hinder integration of mental health into PHC in Uganda.

**Methods:**

A qualitative research design was adopted at national and district level. A total of 18 Key informant interviews were conducted at both levels. Content thematic analysis was the main method of data analysis.

**Findings:**

There were positive gains in working on relevant laws and policies. However, both the mental health law and policy are still in draft form. There is also increased responsiveness/participation of key stakeholders; especially at national level in the planning and budgeting for mental health services. This however seems to be a challenge at both district and community level. In terms of efficiency, human resources, finances, medicines and technologies constitute a major drawback to the integration of mental health into PHC. Ethics, oversight, information and monitoring functions though reported to be in place, become weaker at the district level than at national level due to limited finances, human resources gaps and limited technical capacity. Other governance related issues are also reported in this study.

**Conclusions:**

There is some progress especially in the legal and policy arena to support integration of mental health into PHC in Uganda. However, adequate resources are still required to facilitate the effective functioning of all governance pillars that make integration of mental health into PHC feasible in Uganda.

## Background

“Governments, donors and philanthropists underestimate the challenges of governance in health care delivery…” [1].

The statement by Lewis [1] is used to set the context of this paper. It summarizes the context of health service delivery in most of the developing countries. Mental health problems are common, with over 25 % of people worldwide developing one or more mental disorders at some point in their life [2, 3]. Mental health problems make a significant contribution to the global burden of disease, as measured by disability-adjusted life years (DALYs) [3–5]. In low and middle income countries, life style changes have resulted into to a dramatic epidemiological shift from communicable to non-communicable diseases (NCDs) [6]. It has been estimated that by 2030, NCDs, including neuropsychiatric disorders, will constitute seven of the 10 leading causes of disease burden globally [6, 7].

The burden of mental illness in Africa is also big and the continent has for over decades been prone to civil conflicts and these (civil conflicts), in addition to other factors (such as a high prevalence of HIV/AIDS, poverty, urbanization etc.) have aggravated the burden of mental illness in the affected areas [8]. The health sector is largely poorly funded in most African countries and mental health is one of the poorly developed areas [8].

In Uganda, mental, neurological and substance use disorders are a major contributor to the public health burden. For example, it has been reported that for the two decades of civil conflict in northern Uganda, the region had one of the highest rates of posttraumatic stress disorder (PTSD) in the world, with one study reporting as many as 54 % of the study population having PTSD symptoms and 67 % having depressive symptoms [8]. In our recent studies in post conflict northern Uganda, the prevalence rate for clinical PTSD and depression was 11.8 and 24.7 % respectively [9]. Other studies conducted in other regions [10–13] also indicate high prevalence rates of common mental disorders in the country.

Integration of mental health into primary health care is one of the suggested strategies to effectively respond to the burden of common mental disorders [4]. It is likely to improve patient outcomes, improve adherence to medication, and reduced health care costs [4, 7, 14]. This strategy has been widely embraced for improving mental health service delivery in low and middle income countries, despite its slow operationalization. In Uganda, the Ministry of Health, with support from WHO and World Vision is implementing the Mental Health Gap Action Programme (mhGAP) in three districts, with the aim of fostering integration of mental health into PHC in the country. There are also a number of research initiatives with the same focus. These among others include, the PRIME study which supports integration of mental health activities in Kamuli district and the EMERALD (emerging mental health systems in low- and middle-income countries project which supports health system’s processes for integration of mental health into PHC at both the district and national levels [15].

There is growing recognition of governance in health as a salient theme on the development agenda of health services globally and locally. Governance has been described as deliberate efforts that are concerned with creating conditions of ordered rule and collective action [16]. It signifies change in the meaning of government; where by government has to embark on new processes of governance-embarking on ordered rule and setting new methods as to how society is governed [16]. Governance in health is a cross-cutting theme that is intimately connected with issues surrounding accountability, and an integral part of several key health systems components. Despite its importance in improving health outcomes, governance remains inadequately monitored and evaluated in health systems in low income countries.

It is also true that some few global studies [17, 18] have been conducted on opportunities and challenges of integrating mental health into PHC. However, we learn little from the global studies since most of their information is aggregated at continental/regional level; hence, they shed little/no information on contextual issues that affect governance in the individual countries being studied.

In Africa, one of the elaborate studies that have been conducted on governance issues that support integration of mental health into PHC is by Marias and Inge [6]. However, this study was conducted in South Africa (a middle income country), with a context quite different from that of Uganda (a low income country). In Uganda, there are a few policy/health systems studies that have been conducted, with some focus on governance issues in mental health service delivery [19, 20]. However, their main focus was on how policy and legislations affect integration of mental health into PHC. In this study, we provide quite a broad range of issues based on the Siddiqi and colleagues [21] governance framework which includes: rule of law, strategic vision, participation and consensus orientation, transparency, responsiveness, equity, effectiveness and efficiency, accountability, intelligence and information, and ethics. By doing so, the study provides a detailed outlook (and baseline information for EMERALD) on governance issues that relate to integration of mental health into PHC in Uganda; than any other study conducted so far in the country.

This study is part of a novel project- EMERALD. The EMERALD project aims at improving mental health outcomes in low- and middle-income countries by enhancing health system performance. Specifically, the project aims to identify key health system barriers to, and solutions for, the scaled-up delivery of mental health services in low- and middle-income countries, and by doing so, improve mental health outcomes in a fair and efficient way [15].

## Methods

### Study design

This was a qualitative research study based on key informant interviews. The results reported in this study are an analysis of data collected using an interview guide whose themes were drawn from the Siddiqi and colleagues [21] governance framework. Nine [9] key informants were purposively selected at national level. These included policy makers/managers at the Uganda Ministry of Health as well as senior staff at the national referral mental hospital. Another nine key informants were also purposively selected in Kamuli district. These included members of the District Health Management Team as well as District Administrators. Kamuli district was purposively chosen because it is the study site for the PRIME (Program for Improving Mental Health Care); another research study supporting integration of mental health into PHC in Uganda. All together, a total of 18 key informants were interviewed.

### Study population

As noted above, our study participants were professionals and senior managers, with more than 5 years experience. The senior managers from Ministry of Health headquarters were from the mental health unit, planning and budgeting departments. The professionals from Butabika National Hospital were psychiatrists and one senior Nursing Officer. From the district, the informants were health program managers, an administrator and health facility managers. The demographic profiles of the respondents are summarized in Table 1 below:

**Table 1:** Demographic profile of the respondents

	Informants’ demographic profile/designation	National level	District level
1	Male	6	7
2	Female	3	2
3	Policy maker/program managers	4	2
4	Psychiatrists	4	
5	Senior nurses	1	1
6	Administrators		1
7	Politicians		2
8	Health facility managers		3

### Instrument

This being a multi-centre study, a shared key informant guide was developed for all the study sites (Uganda, Ethiopia, Nigeria, Nepal, South Africa and India). Development of this interview guide was informed by the Siddiqi and colleagues [21] governance framework (see Table 2). Each study site then used the tool as an interview guide. Each interview lasted at least an hour.

**Table 2:** Siddiqi et al. [21] governance framework principles (Adapted from Siddiqi et al. [21])

Principle	Domains
Strategic vision	
Leaders have a broad and long-term perspective on health and human development, along with a sense of strategic directions for such development. There is also an understanding of the historical, cultural and social complexities in which that perspective is grounded	Long-term vision; comprehensive development strategy including health
Participation and consensus orientation	
All men and women should have a voice in decision-making for health, either directly or through legitimate intermediate institutions that represent their interests. Such broad participation is built on freedom of association and speech, as well as capacities to participate constructively. Good governance of the health system mediates differing interests to reach a broad consensus on what is in the best interests of the group and, where possible, on health policies and procedures	Participation in decision-making process; stakeholder identification and voice
Rule of law	
Legal frameworks pertaining to health should be fair and enforced impartially, particularly the laws on human rights related to health	Legislative process; interpretation of legislation to regulation and policy; enforcement of laws and regulations
Transparency	
Transparency is built on the free flow of information for all health matters. Processes, institutions and information should be directly accessible to those concerned with them, and enough information is provided to understand and monitor health matters	Transparency in decision-making; transparency in allocation of resources
Responsiveness	
Institutions and processes should try to serve all stakeholders to ensure that the policies and programs are responsive to the health and non-health needs of its users	Response to population health needs; response to regional local health needs
Equity	
All men and women should have opportunities to improve or maintain their health and well-being	Equity in access to care; fair financing of health care; disparities in health
Effectiveness and efficiency	
Processes and institutions should produce results that meet population needs and influence health outcomes while making the best use of resources capacity for implementation	Quality of human resources; communication processes
Accountability	
Decision-makers in government, the private sector and civil society organizations involved in health are accountable to the public, as well as to institutional stakeholders. This accountability differs depending on the organization and whether the decision is internal or external to an organization	Accountability: internal; accountability: external
Intelligence and information	
Intelligence and information are essential for a good understanding of health system, without which it is not possible to provide evidence for informed decisions that influences the behaviour of different interest groups that support, or at least do not conflict with, the strategic vision for health	Information: generation, collection, analysis, dissemination
Ethics	
The commonly accepted principles of health care ethics include respect for autonomy, nonmaleficence, beneficence and justice. Health care ethics, which includes ethics in health research, is important to safeguard the interest and the rights of the patients	Principles of bioethics; health care and research ethics

### Data analysis

Content thematic analysis was adopted, as the categories that were used during data analysis were drawn from Siddiqi and colleagues [21] governance framework. Content thematic analysis was deemed the most appropriate approach to data analysis because it allows analysis of data using priori analysis categories. During analysis, we related our codes to the Siddiqi and colleagues framework [21]. Under each category (e.g. policy, legislation), themes and subthemes were developed. This same process was repeated for the rest of principles in the governance framework by Siddiqi and colleagues [21].

### Ethics

Ethical approval was secured from Makerere University School of Medicine Research and Ethics Committee. Further ethical approval was secured from Kamuli district health office. All the study objectives were explained to the informants before data collection. Informed consent was obtained from the key informants at both the district and national level. Confidentiality was observed by keeping the data under key and lock and only available to the research team. Informants’ names are not used while reporting results. Their titles are also not included while reporting results to ensure more confidentiality.

## Results

### Strategic direction

Under strategic direction, the focus of this paper is on two vital elements: (a) existing policy frameworks on mental health, (b) legislations on mental health.

### Mental health policies

Uganda has a draft national policy on mental, neurological and substance abuse services [22]. However, this policy has remained in draft form for over 9 years, though it has been a major guiding document in providing the above mentioned services. The country relies on other related health policies such as the Uganda National Health Policy [23] and, the health sector strategic and investment plan (HSSIP) [24] to deliver mental health services. In these two institutional tools, mental health is emphasized as one of the components of the minimum health care package. By being part of the minimum health care package, there are opportunities for inclusion of mental health within PHC activities in Uganda. Mental health is also part of the Uganda National Development Plan [25], the overall national planning framework in Uganda.

“…The mental health system[s] are part of the central ministry of health systems … because mental health is part of the basic minimum package of care, so all plans … and policies coming from the Ministry of Health are by design supposed to have a component of mental health” (Senior Official, Uganda Ministry of Health, Mental Health Unit).

“…At policy level, they mention it explicitly. For example in the minimum health care package it is mentioned explicitly that mental health care is one of the basic eleven – I think they are now thirteen interventions that are provided at all levels of health care. So that is a requirement by policy. And then of course the practice, that is where the variation occurs, based on availability of skills and attitudes. But at policy level that is what we use” (Consultant Psychiatrist, Butabika National Referral Psychiatric Hospital).

The Mental Health Unit at the Uganda Ministry of Health developed a draft strategic plan [26] which was revised in 2012. Just like the Mental Health Policy, the strategic plan is still in draft form. However, despite being a draft, it still guides the planning and delivery of mental health services in Uganda; including integration of mental health into PHC. Thus, we deal with the challenges of not having a fully fledged policy in our discussion section.

With regard to policy framework(s) at the district level, service delivery at this level follows the national policies; and in this case the draft National Policy on mental, neurological and substance abuse services [22]. This draft policy was however unfamiliar (during data collection) to the health workers in the district, including health managers; mainly because it had not been widely disseminated. Integration of mental health into (PHC) was however cited as one of the key policy recommendations that district health systems are struggling to implement under a decentralized system of service delivery.

“…You see, we don’t come up with our own policies…we work under government and government policies. You have to act in accordance with the government policy. Yes, the health managers may know that the policy exists, but what does the policy say? (District Health Manager).

Under the decentralized system of service delivery, districts align their strategic plans with the national strategic plan. In line with this, the district has a strategic and development plan running for 5 years and mental health is expected to be addressed within this plan as an integral component of PHC. However, a number of respondents affirmed that mental health is a neglected area that has no clear budget line in the district plans. It also emerged that the planning process at district level is partly affected and influenced by the available partners and their interests. The planners tend to prioritize areas that are known to be more appealing or in line with the partners’ interests and thus expected to easily attract funding.

“….Decentralization can impact on our service delivery in such a way that we plan for what we want. But our planning is affected by available resources. Some of those areas may be important, but the limited resource envelope doesn’t allow” (District Council Secretary of Health).

“…Yah, we make our priorities. And because different districts have different partners, they plan differently. You can’t say I will plan the same as Mayuge [a neighboring district]. Mayuge has SUSTAIN [a nongovernmental] programme. For me, I don’t have SUSTAIN. So, for me, in HIV/AIDS, I may plan differently because I don’t have that partner” (District Health Officer).

There are also efforts though still in a few areas, to improve the planning capacity of the local governments to deliver mental health services. In Kamuli district, the PRIME project has supported the health managers to develop a district mental health care plan. The lessons learnt are supposed to inform the Uganda Ministry of Health in scaling up integrated mental health care planning and service delivery [19]. World vision Uganda, through the WHO Mental Health Gap Action Programme (mhGAP) is also expanding the delivery of mental health at PHC level in three pilot districts in Uganda. Part of the mhGAP activities is capacity building. The overall aim of the mhGAP intervention in Uganda is that mental health services will be integrated in the national health care plan and implemented at all levels of PHC for the improved wellbeing of the general population in Uganda. Despite the above mentioned efforts in fostering integration of mental health into PHC, there are still challenges in operationalising the plans even where integration of mental health into PHC has been supported.

“…In most discussions already the talk is that we need to scale up these activities. But, our biggest constraint is normally resources. [And] sometimes even technical capability in the local governments. Because they [the local governments] are the key pillars in service delivery. But things are like, do we have many mental health specialists in local government? I don’t think. So that is the one of our challenges in scaling up” (Senior Official, Budgeting Division, and Uganda Ministry of Health).

### Rule of law

Mental health laws in Uganda are currently out of date. A mental treatment Ordinance, to address legal aspects for management and protection of persons with mental illness and the community was developed in 1935. It subsequently became a law (the Uganda mental health treatment law) in 1964. The law was not revised until 2011 when the current mental health bill was produced. To date, this revised mental health bill has not been passed into law. The country therefore largely depends on obsolete legislations and they seem to be too narrow to protect and promote care for people with mental illness in the rapidly changing global and national context. They are also quite deficient in promoting prevention activities in mental health.

“…see, we are largely behind in terms of legislations as the country depends on old Laws written in colonial times. In such a situation, issues to do with patient rights are difficult to enforce; and it is a challenge to some of us who work in the forensic field” (consultant psychiatrist Butabika national referral and teaching hospital).

While service delivery across the country is governed by such national policies and laws, the managers were aware of the fact that districts have some autonomy and may choose to pass bylaws where need arises. With the national mental health law yet to be enacted, the health workers do what is deemed right, offering mental health services apparently in consonance with some of the provisions in the proposed mental health bill. One of the major limitations however was that the proposed mental health law has not been widely publicized.

“…of course we work within those lead national documents. We ensure that we plan within the national objectives. And to some extent, we cannot say it is central. For example we can choose to pass a bylaw here at the district. Is that centralization? No” (District Health Officer).

“…although we have not looked at the national mental health law, I think we are providing services according to the national health policy and law. We cannot operate outside the law. Otherwise, we would have serious problems” (Health facility manager).

### Responsiveness and integration

In this study, we looked at responsiveness at national level, district level and community level. It was noted that political commitment to mental health is largely expressed in the policy documents. However, many of the policy aspirations have largely not been translated into action. Priority is still given to communicable diseases such as HIV/AIDS, tuberculosis, malaria among others. At National level, the planning and budgeting model is based on the *Standard Unit of Output*- the number of patients seen in a given year by a health facility (including a hospital). In essence, diseases that contribute to high morbidity and mortality rates attract more resources than those that are associated with low mortality and morbidity rates. Hence, more resources are allocated to communicable diseases, given their role in contributing to high morbidity and mortality in the country.

“…money is allocated based on the priorities which have been presented by the technical working groups. [There is the] standard units of output. Standard unit is like how many patients they [health facilities] handle in a year. And also of course what type of services they offer. So, money is allocated basing on those kind of approaches and principles. It is not a guess” (Senior Official, Budgeting Division, Uganda Ministry of Health).

“…The pattern [in allocation of resources] was based on mortality and that is it. So even as mortality reduces [for other conditions] it doesn’t influence [planning], we continue with that pattern. Allocation is in the interest of donors, who specifically allocate funds to certain sectors. And then the internal revenue has to fall in certain areas which already exist. So that is just how our budget process takes place” (Consultant Psychiatrist, Butabika National Hospital).

Importantly, it was noted that health is also a political issue in Uganda. The politicians focus more on conditions that affect the majority of the population for political expedience. Conditions that are perceived as not affecting a large part of the population are not given political attention and hence small budgets. There is limited data on epidemiology of common mental disorders in the country and this is likely to affect efforts to put mental health on the policy maker’s agenda.

“… if the politicians are not in support of your ideas you may not get the money you want. So it is whether it is there in the strategic plan. And it is a top priority of the senior top management which is chaired by the minister [the political head of the ministry]. And, whether there is evidence … evidence base for the need for additional resources” (Senior Official, Budgeting Division, and Uganda Ministry of Health).

“…At the district level, the same model is used. The diseases that contribute to high morbidity and mortality are given more priority than those that are associated with low morbidity and mortality such as mental illness…They have resources [districts] but not sufficient. We still have problems of you know maternal deaths, child mortality, which they are concentrating on more. They are not looking at things like malaria because the resources are insufficient -they concentrate on the key concerns of society; which currently is maternal health and child health. But of course mental health is becoming a big problem” (Senior Official, Budgeting Division, Uganda Ministry of Health).

It was further noted that apparently, there seems to be some silence on mental illness in the country. The districts in northern Uganda, which are reported to have a reasonably high burden of mental illness because of the civil strife for over two decades have district plans and budgets not prioritizing mental health as a public health issue. The probable silence apparently negatively affects the responsiveness of both government and private sector institutions to deliver mental health services.

“…There is low demand for mental health services because the public has a different orientation. People don’t complain of symptoms of mental illness. And even when it is severe, they seek alternative methods of healing [traditional healers]. So the demand removes the fact that there is need. So at the health unit level it is as if there is no need” (Consultant psychiatrist, Butabika national referral psychiatric hospital).

The informants consequently expressed the need for community awareness to increase the demand for mental health services in the country. Even in the context of decentralization, responsiveness of health delivery systems to the mental health needs of the general population is still a daunting challenge. Some informants argued that the concept of decentralization is still theoretical, with power, decision making and resource allocation still largely centralized.

“…now when you talk about decentralization, like now when you talk of these drugs… NMS [National Medical Stores] is just sending. And the Ministry [of Health] is aware. That means we do not have powers over it. It is them to decide for us. So, that one is in the center. You can’t call it decentralization when we don’t have power over certain things” (District Council, Secretary for Health).

The situation was reported to be further complicated by the fact that health workers tend to prioritize programs/activities that come with some incentives and disregard or pay less attention to others.

At community level, most districts were reported to be hardly having any psychiatric services. Related services (psychosocial services) are delivered by non-governmental organizations. However, they are poorly funded, uncoordinated and largely not effective.

### Effectiveness and efficiency

The governance issues of interest to this study, under this domain are: human resources, finances, infrastructure, medicines and technologies.

#### Human resources

Human resources are a critical resource in the operationalization of health plans. They are key (human resources) at all levels in supporting the integration of mental health services at PHC level. As noted above, the planning models used in Uganda for planning and modeling affect the human resources available to deliver a particular service.

Despite the small numbers generally, the overall total number of health workers specializing in mental health was said to have increased over the past years.

“…Given where we are coming from, personally am happy that something is coming up at the district level. Because before, mental health workers were not looked at as people that were required by the districts but now there is some positive trend, they have started recruiting mental health workers; they have stated with Psychiatric nurses. They are also now taking up some PCOs [psychiatric clinical officers], although it is at the regional level. So that is really positive” (Manager/Senior Consultant Psychiatrist).

The informants affirmed that the current staffing levels do not conform to the Uganda Ministry of Health staffing norms. They emphasized the need for a well streamlined health management information system for mental health to provide evidence of the need for better human resource management for mental health.

“…No. of course you see, like there are policies providing for human resource at various levels. Then you find given the situation we are in you can’t even fully compare with what is provided for in the law to what is actually on the ground” (Manager/Senior Consultant Psychiatrist).

“…We need proper data… on depression, suicide cases and all the other mental health problems. Do we have that data? No. So, if we don’t have a health management information system that covers mental health, no one will ever plan for human resource for mental health” (Senior Health Official, Planning Unit, Uganda Ministry of Health).

The shortage of human resources for mental health care was partly attributed to mental health related stigma, which is not only extended to the patients, but to the mental health profession in general.

The situation is further complicated by the heavy workloads at the health facilities, which often lead to deployment of the mental health staff to work as general health workers.

“…I think the only thing they are facing at that higher level – national referral and regional referral – is low staffing. Even the psychiatric nurses, the managers of those health units tend to want to allocate them to other duties, not just mental health.” (Consultant Psychiatrist, Butabika National Referral Psychiatric Hospital).

At the district level, one of the notable key barriers to health care was the shortage of human resources to deliver health care. This is largely attributed to the ban on recruitment of health workers, imposed by the central government due to financial constraints. The health managers regretted government’s failure to implement the recommended strategies for attraction and retention of health workers in the rural settings. Although the staff turnover was reported to be relatively low, the medical doctors in particular were described as “a hard to retain category”.

“…many times you hear within the corridors how the health workers are so de-motivated and how they might leave any time. Government brought the top-up allowance scheme for Medical Officers at Health Centre IVs but it has not been consistent in its implementation. They got for some time [top-up allowance], but now they are not getting. It is still a big challenge. And we have tried to functionalize these health centers with theatres but still, there is no one to work in them” (District Health Officer).

Mental health professionals at district level were reported to be very few and not receiving any support in terms of capacity building. Just like at the national level, the situation is further complicated by the fact that some of these specialists are recruited and deployed as general health workers.

“…One practical thing is what we witnessed in Kamuli recently when we went for a visit. Three people were trained but two were removed because other wards were very acute, and they remained with one. But also they say the kind of work they have, they cannot even handle mental health patients because it requires a lot of time. So they have to send that patient to the only one [health worker] left there. That if you start on a mental health patient, you can’t finish” (Senior Consultant Psychiatrist, Butabika National Referral Psychiatric Hospital).

The only category of health workers (though not on the structure of government paid workers) where districts have large numbers are the community health workers. However, these ones show less motivation and enthusiasm towards service delivery because they are not remunerated.

#### Financing

At national level, there are budget conferences. These are used to set priorities and advise on the allocation of resources to all sectors in the Uganda Ministry of Health. The budget conferences are attended by the various stakeholders including the private sector.

The budget conference scrutinizes the plans and budgets and where there are omissions in a sector, they are incorporated. However, due the overwhelming health needs, each sector has to work within a particular budget limit.

Funding for health activities in the district was noted to be through PHC funding. This arrangement provides for a lump-sum of money for the health budget, which is apportioned to different health activities and programs based on the district priorities. In view of the inadequate funding and competing priorities, less prioritized programs such as mental health care receive minimal support from the PHC fund.

“…and we have to prioritize, because funding is not enough. If you put mental health, UNEPI (referring to immunization), reproductive health, compound maintenance etc., mental health might be the last to be thought of” (Senior official, Nursing department).

Such low prioritization of mental health care is largely attributed to poor appreciation of the burden of mental illness and the belief that mental illness is not associated with mortality.

There exists a parallel funding mechanism under which a few partners bring in funds tagged to their specific priority areas; but so far, no partner had funded mental health activities.

#### Infrastructure

Infrastructure for mental health was reportedly available at national level and regional level. Uganda benefited from some project (Support to the Health Sector Plan project, 2001–2006) for strengthening mental health systems. At national level, Butabika National Referral Psychiatric hospital was renovated. In each region, a metal health unit was constructed. However, this is not the case at district level. It was noted that the district is under-resourced in terms of infrastructure, with most of the facilities having insufficient space to provide various services. At some of the health facilities, the staff can hardly find space and privacy to deliver mental health services. A few health facilities had however benefited from support and funding from some external partner(s) for infrastructural development.

“…We are seeing them at OPD with the others. The problem is space. Because even this office here, it is where the in-charge sits; and at times it is where he sees patients. We even wanted to make particular clinic days, but we have shortage of where to see them. Even those who need admission, we don’t have a mental health ward” (Psychiatric Nurse at Health Centre IV, Kamuli district).

### Medicines and technologies

It is apparently clear that the system for purchase and distribution of medicines depends on the disease patterns, as reported in the health management information system data. However, nearly all respondents at district level commented on the frequent stock out of medicines, and observed that all health facilities complain of delays and an inadequate supply of medicines. The situation was reported to be particularly worse for mental health medicines. The current medicines supply system (termed “push system”) was reported to be problematic, giving the health facilities less autonomy on the type and quantity of medicines to be supplied. Under this system, the responsible authority (national medical stores) determines what to supply, when, and in what quantities. This was reported to be one of the major challenges affecting service delivery.

“…that push method is not good…and that thing is affecting service delivery. Because sometimes they just push medicines and even things (supplies) which are not needed down there” (Senior Official, Nursing department).

Most respondents believed that the medicines supply system needs to be revised so that health facilities have the autonomy to request for the medicines they need, based on the demand.

“…sometimes we take 2 months without drugs in our health facilities. Then also the supplies do not depend on the demand. For example, you may find that for facility A, they bring 5 boxes of condoms and 2 boxes of coartem, and yet the condoms are not so much useful compared to coartem. And more-over, they don’t deliver in time” (District Secretary for Health).

### Participation and collaboration

The process of developing the strategic plans involved quite wide consultations with key stakeholders. The draft mental health strategic plan provides guidelines for quarterly and annual planning for the mental health unit. All these processes are undertaken by all units and departments at the Uganda Ministry of Health.

“…Every quarter […] there is an annual performance review meeting where stakeholders from all levels of care and all partners and other relevant sectors participate. They examine the reports for the previous years and help to set priorities for the coming year…. the stakeholders for mental health must look at, evaluate what has happened in the past and seek priorities for the coming period. So it is part of the planning process of the Ministry of Health and this goes down through all the levels up to district level”. (Senior Official, Uganda Ministry of Health, Mental Health Unit).

“…So the coordination is done at national level to involve other sectors and when there are agreed decisions they are communicated to the district level. But at district level there is a process for coordination which is done through the technical meetings of the different sectors because there is already an established structure for that at the district level. And these other sectors at the district level also receive communication from their sectors from the national level” (Senior Official Uganda Ministry of Health, Mental Health Unit).

The district health department was reported to benefit from some multi-sectoral collaboration and involvement of stakeholders, mostly through the Technical Planning Committee (TPC). This was however, reported to apply for other health programs other than mental health. The TPC has membership of stakeholders/heads of various departments and harnesses plans from various departments into a single district strategic plan. The minimal collaboration and involvement for mental health was partly attributed to the limited awareness and appreciation of mental health, and the magnitude of the problem of mental illness in the community. Much of the collaboration and partnership was specifically reported to be in the field of management of HIV/AIDS. Some of the respondents could hardly comment on multi-sectoral collaboration for mental health as they believed it was non-existent.

Furthermore, at district level, there were no reports of service user involvement in planning, supervision and monitoring of mental health services, as expressed by one key informant below:

“…To a lesser extent. Again by policy, it is a requirement, that there is representation. But that representation is very thin. For example there is one member who represents interests of persons with mental illness on the boards – for example here at the national mental referral hospital. But then as you go to regional hospitals there is no representation and so on. So it gets lost eventually although it is in the policy instruments. And then those people who sit there have a weak voice. We know that service users get heard when they are many, when it is one, they get ignored as a sick person. That is an area where hopefully we can get support from civil societies, strengthen it, and then things will fall in place, perhaps” (Consultant Psychiatrist, Butabika National Referral Psychiatric Hospital).

### Equity and inclusiveness

Our focus here was whether the health service delivery systems provide for opportunities to special groups. At the national level, equity was reportedly achieved through budget allocations.

“…As long as the demand is expressed. The system being a public system and being supported by government is not a rigid system. Any time requests for change, say in medicine policy and access to different services can be done. So that there is an opportunity for people to share resources from different levels. Either upwards or downwards depending on whether you are at district level or you are at the lowest health facility” (Senior Official, Mental Health Unit).

“…I think it is. I think it is because districts as I said are grouped according to their poverty level, to their population. Because usually calculations are done per capita. But also they look at other … how vulnerable … how many … if they have like a district which is prone to epidemics then these considerations are factored in the formula when the funds are being disbursed” (Senior Official, Uganda Ministry of Health, Mental Health Unit).

Mental health being a neglected area, it has not benefited from such budget arrangements. It emerged that while there is free access to health care at all government health facilities, there is a disparity in access to care by location, gender and age. Apparently, the major consumers of health care services are women and children.

“…when you look at our communities, you find that its women and children who mostly come to health facilities. I can say for every 10 patients you find at a health facility, about 8 will be women and children. The men don’t want to come for treatment. They pretend to be o.k, and others just buy drugs” (General health worker).

### Ethics and oversight

The key elements here were accreditation of health workers, ethics in practice and research. Informants were aware of the vital importance of accreditation but this has not been largely implemented in the country.

“…It’s [accreditation] something that we’ve seen work very well in many places and I think it’s something we should begin to advocate for. Unfortunately accreditation has not been taken very seriously, but I think given the interactions we are now having with the international colleagues, I think that’s the direction that really needs to be taken” (Senior Manager Butabika National Referral and Teaching Hospital).

There were also concerns that most of the trainings lack accreditation and have low standards. In terms of enforcement of research ethics, the country was reported to have a number of review boards, and the Ministry of Health is interested in enforcing ethical standards.

“…I cannot know… I cannot say that I know whether [ethics] are followed to the latter. But I think generally there is good information for the people and researchers within the country about maintaining the ethics. And that is why all researchers… they are required to go to the institutions of learning to get the IRBs and also to the National Council of Science and Technology. And the ministry of health also has a requirement, which unfortunately is flouted by some researchers, that no research should be done in a programme area, unless there has been consultation with that programme to make sure what is going to be done is actually ethical” (Senior Official Uganda Ministry of Health, Mental Health Unit).)

### Intelligence and information

Implementation of mental health policies was reported to be monitored through support supervision (both for technical support supervision and the general support supervision).

“…Technical support visits are used to monitor and collect information on program performance. If you are lucky and somebody decides to do research in a certain area which relates to that… but also we get feedback from users… from the users, because civil society organizations which are also user organizations have informed the users on what to expect from the health system. So usually they report … they can report through these formal meetings but they can also write reports where there are serious situations which they want to be addressed” (Senior Official, Uganda Ministry of Health, Mental Health Unit).

It was noted that the district follows the national health management information system (HMIS). The HMIS forms include a number of indicators for various health problems. Data is compiled by records staff at individual health facilities and reported through their managers to the responsible HMIS coordination office on a monthly basis. Such data is basically important for planning purposes. It however emerged that the system has a number of flaws such as absence of a mechanism for verification of the data. In an informal exchange with some health workers, it emerged that often there are irregularities and inconsistencies in the information collected; and they were skeptical over use of such data for planning.

Respondents further commented on the weak implementation of mechanisms for monitoring and evaluation of the health programs. It was noted that very little monitoring and support supervision goes on at health facilities; partly due to inadequate resources and absence of specialists.

### Accountability and transparency

Accountability and transparence at national level are enforced through regular audits, support supervision and regular reviews (quarterly and annual). At the regional level, accountability is enforced through the same avenues such as hospital boards, which provide oversight functions.

The district has weak mechanisms to ensure accountability and transparency in service delivery; partly attributable to the inadequate monitoring and supervision of health workers. Some of the health workers also cited poor coordination and communication between the health workers, health facility managers and the Ministry of Health headquarters; which apparently antagonizes service delivery.

## Discussion

It has been reported that neuropsychiatric disorders are estimated to contribute to 13 percent of the global burden of disease [27]. It is also true that between 76 and 85 % of people with severe mental disorders receive no treatment for their disorder in low-income and middle-income countries [5]. Despite the high burden of mental health problems, there is paucity of information worldwide on the availability of mental health resources to respond to this burden [2, 5, 27]. Recent research also indicates that the available mental health resources are not well distributed across countries [5, 27]. In addition, mental health resources are more limited in Africa as compared to other continents. Unless governance issues are addressed in health systems development, many countries in Africa are likely not to achieve the MDGs [28].

This paper has focused on governance issues relating to integration of mental health into PHC. We have provided information on quite a wide range of governance pillars; which areas have not been largely tackled in previous studies in Uganda. Previous studies have largely focused on policy and legislative issues. Overall, the findings indicate some level of progress on some governance pillars (issues) but at the same time stagnation in others areas on the same pillar. However, this seems not unique to Uganda as many other countries elsewhere, are into a similar struggle, especially in low income countries [1, 5, 28].

It has been observed that governance is the most complex and a critical building block of any health system [26]. Government in essence should play a critical role in promoting governance in the health sector through its rules and systems [28]. It has been further observed that it (governance) encompasses the role of the government in health care and its relationship to other actors whose activities impact on health [26]. It represents a continued process involving different components, from policy and legislation formation to financing and monitoring. In order to improve governance, the reforms must be fundamental and sustainable. The reforms should be sustained for a long time based on the fact that major reforms would have taken place in the health delivery system. The approaches to policy reform should also be coherent [29, 30]. In our discussion below we focus on several pillars of governance.

In terms of legislation, there is increasing interest globally in mental health and human rights. In this respect, our findings indicate that Uganda has made some progress in the legislative arena by developing a dedicated Mental Health Law. Unfortunately, this piece of legislation has remained a draft for a long time indicating some level of stagnation in the same area. There is however a possibility that Uganda is transiting from obsolete to new legislations that match with the changing context in the country. It is also clear that quite a number of countries in Africa are working on new legislations [28]. Other studies have also noted that the process of developing new legislations has been slow in Africa [26]. Yet, across the continent these legislations are vital in supporting integration of mental health services into PHC level. Only 15 percent of the study countries in Africa had initiated or revised their legislations in 2005 or later [27]. The mental health bill should be expedited in Uganda to fill this gap. Uganda should learn from South Africa which has an acted Mental Health Act [31] and has been instrumental in advancing human rights issues in the country [7]. The enacted legislation (the Uganda Mental Health Act) should then be widely disseminated and adequate resources allocated for its implementation [27]. In addition, relevant government departments need to be trained to implement this legislation [7] as dedicated mental health legislation does not automatically translate into quality services [27].

Mental health policies and plans are essential tools in governance of health services [27]. As observed, mental health plans serve to delineate strategies and activities that will be implemented to meet policy objectives and typically specify elements such as budget and a time frame for implementation [27]. And, they play a critical role in translating policy into practice [27]. WHO argues countries to define and implement mental health policies and plans in all regions in the world [27]. This study has also indicated that Uganda has quite a number of policies that support planning and delivery of mental health services. However, the dedicated (specific) mental health policy is still in draft form. There are quite a number of challenges associated with this; the country cannot easily attract resources in the absence of a full policy and it becomes difficult to define the required and stable structure needed to deliver mental health services (including the requirements at PHC level). There are lessons to learn from South Africa which has a Mental Health Policy Framework and strategic Plan [32] -which are fully developed and quite functional.

In terms of responsiveness, our findings indicate that there are avenues to foster participation of key stakeholders in the health sector in Uganda. There is regular interaction between policy makers at the Uganda Ministry of Health and key partners in the health/mental health sector. There are also opportunities for sharing and review of quarterly and annual plans by policy makers at the Uganda Ministry of Health and the key stakeholders. The only challenge is that most of the NGOs operating in the mental health field are quite small and under resourced to engage in effective community consultation before they can represent the civil society in planning and budgeting process at national and district levels. The NGOs also have limited geographical scope. It would be important that the few existing NGOs working in the mental health field are availed adequate resources to undertake effective planning processes.

It has been argued that without adequate financing, mental health policies and plans remain only in the realm of good intentions [27]. The challenge however is that in making decisions on health funding in most low income countries, the donors and policy makers base their decisions on epidemiological evidence that indicates the burden of disease to the population [4]. The absence of epidemiological data on common mental disorders in Uganda negatively affects the planning for mental health services [10].

WHO recommends decentralization of mental health resources so that treatment is shifted from institutionalized care in mental hospitals to community-based care [27]. Decentralization is meant to bring the much needed services closer to the people. Institutionalized care becomes expensive as compared to community based care. However, in Uganda, most informants were not feeling the impact of decentralization. Based on the informant’s views, the decentralization policy seems not to be adequately supported with resources to improve service delivery as more resources are spent on institutional care.

In terms of effectiveness and efficiency, our focus was on human resources, infrastructure and medicines. It has been noted that the number of specialized and general health workers dealing with mental health in low-income and middle-income countries is grossly insufficient [5, 28]. WHO also notes that almost half of the world’s population lives in countries where, on average, there is one psychiatrist to serve 200, 000 or more people, and, other mental health care providers who are trained in the use of psychosocial interventions are even scarcer (such as social workers and psychologists). Yet, mental health personnel are the most valuable resource within the mental health system [27]. Our findings indicate that there were attempts in Uganda to improve the number of health workers through recruitment and training. However, lower level health units are still not covered especially at the district level. The lack of adequate workforce creates a barrier to the integration of health interventions into PHC [29]. It was also reportedly hard to retain skilled manpower in the mental health sector at this level. In addition to the above, many of the health workers at the various levels of the national health systems in Africa were not trained to lead and govern [28]. They were largely trained to prevent and control diseases/health conditions [28]. Hence, the urgency to revise the curriculum of schools of public health, medical schools and, other schools of health sciences [28]. A similar challenge is reported in Ethiopia [1]. Bridging this gap requires training professionals from other medical fields [26]. However, stigma and low motivation to work in the mental health sector defeats some of these efforts. And, globally the number of mental health professional especially graduating from institutions is small [27]. Though infrastructure for mental health has improved in Uganda in the recent past, at the district level, there is still limited infrastructure to deliver quality mental health services. Lack of drugs contributes to low utilization of health services [1]. Availability of basic medicines for mental disorders in (PHC) is notably low (in comparison to medicines available for infectious diseases and even other non-communicable diseases), and their use restricted because of the lack of qualified health workers with the appropriate authority to prescribe medications in low income countries such as Uganda [5]. This is a problem at both national and lower level health units. Priority seems to be given to communicable diseases given their role in contributing to morbidity and mortality. Studies conducted elsewhere in Africa (Nigeria and Ethiopia) indicate a similar challenge [1].

It has also been argued that governance is not possible without monitoring and evaluation [27]. It has been further noted that the primary purpose of a mental health information system is not simply to gather data, but rather to enable decision-making that will lead to more effective governance and service improvement [27]. For example, utilization data and patient satisfaction offer complementary metrics of health system effectiveness because under-utilized public facilities or the dissatisfaction of target groups often point to implementation problems [1]. By monitoring these measures, it is possible to determine how seriously governance woes are affecting a health system and correctional measures undertaken [1]. Our findings indicate limited capacity to undertake effective monitoring and evaluation systems (through the HIMS) especially at district level. The data in the HIMS at this level was reportedly poor and not reliable for use. Programmatic efforts need to be undertaken to rectify this limitation in governance.

Accreditation is starting to be appreciated for all health professionals but adequate resources need to be available to institutions that enforce ethics. This also applies to research and ethics institutions in Uganda.

The overall limitation in this study is that most informants were very busy (with government programs) and some interview sessions could be broken. However, follow up sessions could be organized to complete the affected interviews.

## Conclusions

We find resonance between our findings and Lewis [1] who noted that the rush to endorse the MDGs and the eagerness to translate these goals into real programs has largely overlooked the limited ability of institutions to deliver health services. As further noted, the need to identify and address weak governance issues in service delivery is often lost in the commitment to raise funds and expand services [1]. Addressing this problem (inequity) will however require fundamental changes in policy reform [29, 30, 33] Mechanisms exist for addressing these types of problems/dilemmas through better management, improved logistics and information systems, and strengthened accountability [1]. However, these mechanisms are rarely employed in Uganda [1]. Governance issues should be addressed in comprehensive, intersectoral, effective and well coordinated mental health care plans to address the burden of mental illness in Uganda and elsewhere in the world [5]. Increasing opportunities for health financing (for example through cost sharing and prepayment mechanisms, [29]) will help in addressing most of the dilemmas that affect the integration of mental health into PHC as discussed above. A mixed system of government, individual and other private resources will go a long way in availing resources to support the integration of mental health into PHC.

## Abbreviations

EMERALD: emerging mental health systems in low- and middle-income countries
LAMICS: low and middle income countries
mhGAP: Mental Health Gap Action Program
PTSD: posttraumatic stress disorder
PHC: primary health care

## References

[1] Lewis M (2006). Tackling healthcare corruption and governance woes in developing countries.

[2] World Health Organization (2011). Department of mental health and substance dependence, non-communicable diseases and mental health.

[3] Thornicroft G, Alem A, Santos RAD, Barley Drake E (2010). World Psychiatry.

[4] Patel V, Kleinman A (2003). Poverty and common mental disorders in developing countries. Bull World Health Organ.

[5] World Health Organization (2003). Department of mental health and substance dependence, non-communicable diseases and mental health.

[6] Marais DL, Petersen I (2015). Health system governance to support integrated mental health care in South Africa: challenges and opportunities. Int J Ment Health Syst.

[7] World Health Organization (2004). The global burden of disease: 2004 update.

[8] Roberts B, Ocaka KF, Browne J, Oyok T, Sondorp E (2008). Factors associated with post- traumatic stress disorder and depression amongst internally displaced persons in northern Uganda. BMC Psychiatry.

[9] Mugisha J, Muyinda H, Malamba S, Kinyanda E (2015). Major depressive disorder seven years after the conflict in northern Uganda: burden, risk factors and impact on outcomes (The Wayo Nero Study). BMC Psychiatry.

[10] Mugisha J, Muyinda H, Wandiembe P, Kinyanda E (2015). Prevalence and factors associated with posttraumatic stress disorder seven years after the conflict in three districts in northern Uganda (The Wayo-Nero Study). BMC Psychiatry.

[11] Kinyanda E, Woodburn P, Tugumisirize J, Kagugube J, Ndyanabangi S, Patel V (2011). Poverty, life events and the risk for depression in Uganda. Soc Psychiatry Psychiatr Epidemiol.

[12] Abbo C. Profiles and outcome of traditional healing practices for severe mental illnesses in two districts of Eastern Uganda. PhD Thesis Karolinska Institute and Makerere University. Stockholm: Kalolinska University; 2009. 10.3402/gha.v4i0.7117 PMC3150106 21845144

[13] Ovuga E, Boardman J, Wasserman D (2005). The prevalence of depression in districts of Uganda. Soc Psychiatry Psychiatr Epidemiol.

[14] World Health Organization (2002). The world health report 2002: reducing risks, promoting healthy life style.

[15] Semrau M, Lempp H, Keynejad R, Evans-Lacko S, Mugisha J, Raja S, Lamichhane J, Alem A, Thornicroft G, Hanlon C (2016). Service user and caregiver involvement in mental health system strengthening in low- and middle-income countries: systematic review. BMC Health Serv Res.

[16] Stoker G (1999). Governance as theory. Five propositions. UNESICO.

[17] Patel V, Belkin GS, Chockalingam A, Cooper J, Saxena S (2013). Grand challenges: integrating mental health services into priority health care platforms. PLoS Med.

[18] World Health Organization (2009). WHO-AIMS mental health systems in selected low- and middle-income countries: a WHO-AIMS cross-national analysis.

[19] Ssebunnya J, Kigozi F, Ndyanabangi S (2014). Mental health law reforms in Uganda: lesson learnt. Int Psychiatry.

[20] Kigozi F, Ssebunnya J, Kizza D, Cooper S, Ndyanabangi S, MHaPP (2010). An overview of Uganda’s mental health care system: results from an assessment using the World Health Organization’s assessment instrument for mental health systems (WHO-AIMS). Int J Ment Health Syst.

[21] Siddiqi S, Masud TI, Nishtar S, Peters DH, Sabri B, Bile KM (2009). Framework for assessing governance of the health system in developing countries: gateway to good governance. Health Policy.

[22] Ministry of Health (2009). Uganda draft national policy on mental, neurological and substance use services.

[23] Ministry of Health (2010). Uganda ministry of health policy.

[24] Ministry of Health (2010). Uganda health sector strategic investment plan.

[25] Uganda National Development Plan (2010). Ministry of finance and economic development.

[26] Ministry of Health (2009). Mental health unit strategic plan (draft). Uganda ministry of health policy.

[27] Morris J, Lora A, McBain R, Sexena S (2012). Global mental health resources and services: a WHO survey of 184 Countries. Public Health Rev.

[28] Kirigia JM, Kirigia DG. The essence of governance in health development. Int Arch Med. 2011;4:11. http://www.intarchmed.com/content/4/1/11. 10.1186/1755-7682-4-11 PMC3072323 21443766

[29] Senkubuge F, Modisenyane M, Bishaw T. Strengthening health systems by health sector reforms. Glob Health Action. 2014;7:23568. http://www.dx.doi.org/10.3402/gha.v7.23568. 10.3402/gha.v7.23568 PMC4651248 24560261

[30] Kaseje D. Health care in Africa: challenges, opportunities and an emerging model for improvement. Paper presented at the Woodrow Wilson international center for scholars. 2006.

[31] Mental Health Act (Act 17 of 2002). Republic of South Africa. 2002.

[32] Department of Health (2013). National mental health policy framework and strategic plan, 2013–2020.

[33] Kirigia JM, Ota MO, Motari M, Bataringaya JE, Mouhouelo P (2015). National health research systems in the WHO African Region: current status and the way forward. Health Res Policy Syst.

